# *Candida* Infections: The Role of Saliva in Oral Health—A Narrative Review

**DOI:** 10.3390/microorganisms13040717

**Published:** 2025-03-23

**Authors:** Riyoko Tamai, Yusuke Kiyoura

**Affiliations:** Department of Oral Medical Science, Ohu University School of Dentistry, 31-1 Misumido, Tomitamachi, Koriyama, Fukushima 963-8611, Japan

**Keywords:** *Candida* species, saliva, oral diseases, biofilm, immunity

## Abstract

*Candida* species, particularly *Candida albicans*, are causative agents of oral infections to which immunocompromised patients are especially susceptible. Reduced saliva flow (xerostomia) can lead to *Candida* overgrowth, as saliva contains antibacterial components such as histatins and β-defensins that inhibit fungal growth and adhesion to the oral mucosa. *Candida* adheres to host tissues, forms biofilms, and secretes enzymes required for tissue invasion and immune evasion. Secretory asparaginyl proteinases (Saps) and candidalysin, a cytolytic peptide toxin, are vital to *Candida* virulence, and agglutinin-like sequence (Als) proteins are crucial for adhesion, invasion, and biofilm formation. *C. albicans* is a risk factor for dental caries and may increase periodontal disease virulence when it coexists with *Porphyromonas gingivalis*. *Candida* infections have been suggested to heighten the risk of oral cancer based on a relationship between *Candida* species and oral squamous cell carcinoma (OSCC) or oral potentially malignant disorder (OPMD). Meanwhile, β-glucan in the *Candida* cell wall has antitumor effects. In addition, *Candida* biofilms protect viruses such as herpesviruses and coxsackieviruses. Understanding the intricate interactions between *Candida* species, host immune responses, and coexisting microbial communities is essential for developing preventive and therapeutic strategies against oral *Candida* infections, particularly in immunocompromised individuals.

## 1. Introduction

*Candida* species are fungi that cause opportunistic infections in immunocompromised patients and can be found even in healthy individuals at sites such as the oral cavity, gastrointestinal tract, and vagina [1,2]. Among *Candida* species, *Candida albicans* is the most prevalent and clinically significant. While *C. albicans* is the most common species found in the oral cavity, non-*albicans Candida* species also play a significant role in oral fungal colonization and infection.

Oral candidiasis, commonly known as oral thrush, is an opportunistic fungal infection primarily caused by *C. albicans* overgrowth in the oral cavity. It is also increasingly associated with non-*albicans Candida* species, particularly in medically compromised patients with a history of multiple azole antifungal treatments [3,4,5]. Oral candidiasis can manifest as acute or chronic infection, with risk factors including impaired salivary function, dentures, high carbohydrate diet, smoking, and immunosuppressive conditions [6]. Management typically involves antifungal treatment, with prophylaxis recommended for high-risk groups. Host immune responses, particularly innate defenses and helper T cell type 17 (Th17) adaptive immunity, play an important role in controlling *Candida* growth and preventing its tissue invasion via the production of interleukin (IL)-17, a proinflammatory cytokine [7]. However, antimicrobial resistance in *Candida* species is an increasing concern in the management of invasive candidiasis [4,8,9]. Resistance mechanisms include efflux pump induction, mutations in the target enzyme of azoles or echinocandins, and mutations in the FKS gene, which encodes 1,3-β-glucan synthase [4,10]. Standardized susceptibility testing methods established by the Clinical and Laboratory Standards Institute (CLSI) and the Anti-Fungal Susceptibility Testing Subcommittee of the European Committee for Antimicrobial Susceptibility Testing (EUCAST) are crucial for detecting resistance and determining clinical breakpoints [11]. Antifungal resistance is associated with higher minimum inhibitory concentrations, poorer clinical outcomes, and breakthrough infections.

*Candida* species have several virulence factors and are able to adhere to host tissues, form biofilms, and produce hydrolytic enzymes. They are also dimorphic, occurring in both yeast and hyphal forms, and the ability to switch forms is essential for fungal tissue invasion and immune evasion. The cell wall of *Candida* species contains complex polysaccharides such as mannans, glucans, and chitins, which play critical roles in immune recognition and response. Thus, *Candida* species are recognized by a number of receptors such as Toll-like receptors (TLRs) and C-type lectin receptors and induce proinflammatory cytokine production in various cell types, including epithelial cells that serve as barriers to oral candidiasis [12]. The outermost layer of the cell wall, which is composed of mannans, defines the serotypes of *Candida* species and interacts with host immune cells to trigger phagocytosis and other immune responses [13,14,15]. Phagocytosis by macrophages induces the production of reactive oxygen species (ROS), which are critical for pathogen killing. However, live *C. albicans* suppresses ROS production in phagocytes. Moreover, macrophages and neutrophils can engulf yeasts but not hyphae [16,17].

**Figure 1:**
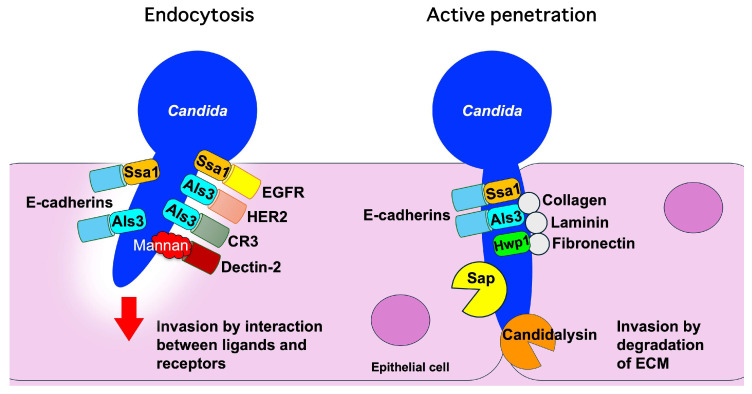
Two primary invasion methods are induced endocytosis and active penetration. Active penetration relies on hyphal elongation and secreted enzymes, such as secreted aspartyl proteinases (Saps), which can degrade interepithelial junctions. Abbreviations: Als, agglutinin-like sequence; EGFR, epidermal growth factor receptor; HER2, human epidermal growth factor receptor 2; CR3, complement receptor 3; Hwp1, hyphal wall protein 1; Sap, secretory aspartyl proteinase; ECM, extracellular matrix.

*Candida* species interact with epithelial cells as both commensals and pathogens, employing various mechanisms for adhesion and invasion [18]. Two primary invasion methods are induced endocytosis and active penetration (Figure 1). Induced endocytosis involves fungal invasins, proteins that facilitate host cell invasion, and includes Als3, which binds to host proteins and triggers clathrin-mediated internalization [18,19]. Active penetration relies on hyphal elongation and secreted hydrolases to breach epithelial barriers [19,20]. *Candida* species can also degrade interepithelial junctions using secreted aspartyl proteinases (Saps) and cause significant epithelial damage [20].

## 2. Candida Albicans and Non-*albicans Candida* Species

*C. albicans* and *C. tropicalis* exhibit distinct characteristics in biofilm formation, virulence, and adhesion mechanisms. *C. albicans* demonstrates greater virulence in normal mice, but *C. tropicalis* is more virulent in immunocompromised mice, particularly when administered orally [21,22]. *C. tropicalis* also demonstrates higher biofilm production and cell viability within biofilms [23] and has the ability to tolerate high salinity and specific metabolic pathways distinguished from other *Candida* species [24,25]. The species is particularly virulent in neutropenic hosts, with a notable frequency of hematogenous spread [26].

*C. albicans* is more prevalent than *C. parapsilosis*, but the latter is more frequently isolated from blood and devices [27]. *C. albicans* infections are associated with higher mortality rates in neonates compared with *C. parapsilosis* [28]. Both species exhibit similar adhesion behavior to silicone catheters [27,28]. Phenotypic analysis revealed shared and unique traits between *C. albicans* and *C. parapsilosis*, particularly in biofilm regulation [29]. *C. parapsilosis* induces different T cell responses in human peripheral blood mononuclear cells compared with *C. albicans*, producing less IL-17 but more IL-10, an anti-inflammatory cytokine [30]. Both species are recognized by dectin-1, a C-type receptor, although there are differences in the production of mitogen-activated protein kinase (MAPK)-dependent cytokines [30].

Differences between *C. albicans* and *C. parapsilosis* are summarized in Table 1.

*C. metapsilosis* and *C. orthopsilosis* are closely related fungal species that were previously grouped together, with *C. parapsilosis* being part of the *C. parapsilosis* complex [31]. *C. metapsilosis* and *C. orthopsilosis* account for 1–10% of infections previously attributed to *C. parapsilosis*. Both are considered less virulent than *C. parapsilosis* sensu stricto [32,33,34]. *C. orthopsilosis* and *C. parapsilosis* exhibit similar adhesion abilities and virulence, while *C. metapsilosis* shows reduced adhesion to human buccal epithelial cells and lower virulence potential in experimental vaginal candidiasis [34]. Molecular identification methods, such as polymerase chain reaction-restriction fragment length polymorphism (PCR-RFLP) of the SADH gene and specific PCR amplification of the RPS0 intron, have been developed to differentiate both species [31,35]. *C. parapsilosis*, *C. metapsilosis*, and *C. orthopsilosis* only show slight differences in antifungal susceptibility, typically with a good susceptibility profile to most antifungal agents [33,36]. Accurate identification is important for understanding the epidemiology and clinical significance of these species.

*C. albicans* and *C. dubliniensis* are closely related pathogenic yeast species, although the former is more pathogenic and prevalent in human infections [37]. The reduced virulence of *C. dubliniensis* is partly due to its limited ability to form hyphae, which is inhibited by nutrients at alkaline pH [38]. Genomic comparisons have revealed an expansion of virulence-related gene families in *C. albicans* and increased pseudogenization in *C. dubliniensis*, suggesting that the latter may be undergoing reductive evolution [37]. It is crucial to accurately identify these species when treating oral candidal infections, as they differ in antimycotic resistance [11]. Phenotypic methods for differentiation include growing them on hypertonic Sabouraud broth and tobacco agar, with *C. dubliniensis* showing growth inhibition on the former and producing characteristic rough, yellowish-brown colonies with abundant hyphae and chlamydospores on the latter [39,40].

*C. auris* is an emerging multidrug-resistant fungal pathogen that has caused significant outbreaks globally with high mortality since being first described in 2009 [41,42,43]. *C. auris* exhibits strain-specific differences in virulence, with some isolates demonstrating pathogenicity comparable to that of *C. albicans* in animal models [41,44]. Unlike *C. albicans*, however, *C. auris* does not produce hyphae and only forms rudimentary pseudohyphae [41]. Multi-omics analyses have revealed significant differences between *C. auris* and *C. albicans* in carbon utilization, lipid content, and protein profiles, which may contribute to the drug resistance and virulence of *C. auris* [42]. Genomic studies have identified five geographically distributed and genetically divergent lineages of *C. auris*, all emerging after 1996 [10]. The unique cell surface mannan of *C. auris* is enriched in β-1,2 linkages and binds strongly to IgG and mannose-binding lectin, potentially contributing to the pathogenesis and colonization abilities of the fungus [45]. *C. auris* demonstrates resistance to fluconazole, although it remains susceptible to salivary histatin 5, a cationic peptide with significant antifungal activity [8]. Compared with *C. albicans*, *C. auris* exhibits higher tolerance to oxidative stress and better survival within neutrophils, suggesting that it is resistant to ROS [8]. Intravenous immunoglobulin (IVIG) therapy has shown promise in preventing and treating *C. auris* infections in mouse models, with IVIG lots containing higher titers of *Candida*-specific IgGs providing better protection [46]. Immunoproteomics studies have identified several immunoreactive proteins in *C. auris* that are recognized by IgGs from infected mice and humans and can potentially serve as diagnostic or therapeutic targets [47]. *C. auris* appears to induce a less robust innate immune response than *C. albicans*, which may be related to differences in cell wall structure [45,48]. The rapid emergence and spread of *C. auris*, coupled with its multidrug resistance, poses a serious challenge to public health systems worldwide.

The emergence of drug-resistant microorganisms has become a major concern worldwide, and *Candida* is no exception. In addition to *C. albicans*, which is the primary cause of *Candida* infections, antifungal drug-resistant non-*albicans Candida* species are becoming increasingly prevalent [8,9], highlighting the importance of accurate species identification and antifungal susceptibility testing for effective patient management and infection control. However, the limited availability of antifungal drugs due to the eukaryotic similarity between fungi and humans further complicates treatment options [11]. Ongoing surveillance and research are essential to address evolving challenges in fungal infections.

## 3. Oral Candidiasis

Oral candidiasis presents in various forms, including pseudomembranous, erythematous, hyperplastic, and angular cheilitis, median rhomboid glossitis, and denture stomatitis [49,50]. These manifestations can occur singly or in combination, influenced by host immune responses and fungal virulence factors. Oral candidiasis is often linked to underlying conditions such as acquired immunodeficiency syndrome (AIDS), diabetes, and immunosuppression and can serve as a clinical marker for significant predisposing conditions [50].

Pseudomembranous candidiasis is associated with local immune breakdown, while erythematous candidiasis may involve hypersensitivity to *Candida* antigens [51]. Pseudomembranous candidiasis manifests clinically as white, removable lesions on oral mucosa and can cause discomfort, pain, and taste alterations [52]. However, erythematous candidiasis presents as red, sometimes ulcerated lesions on the oral mucosa, often accompanied by a burning sensation and metallic taste [53]. This form of candidiasis is as serious a prognostic indicator for AIDS progression as the more recognizable pseudomembranous type [54]. Erythematous candidiasis can also occur in patients with diabetes mellitus due to high glucose levels in oral fluids and decreased immunity. Other predisposing factors include dentures, reduced salivary flow, and broad-spectrum antibiotic use [53]. While pseudomembranous candidiasis is linked to lower CD4^+^ cell counts, erythematous candidiasis is associated with high viral load [55]. Histopathologically, both forms show a marked reduction in CD4^+^ cells, with pseudomembranous candidiasis exhibiting a more pronounced local immune response breakdown and erythematous candidiasis demonstrating a hypersensitivity reaction to *Candida* antigens [51]. Risk factors for these variants differ, suggesting distinct pathogenic mechanisms [55].

Hyperplastic candidiasis represents a superficial cellular reaction against the pathogen and manifests as white patches on the oral mucosa [56,57]. Hyperplastic candidiasis lesions are typically asymptomatic and regress with antifungal therapy, but untreated cases may develop into carcinomas. The pathogenesis involves a complex interplay between fungal virulence factors and host immune responses, with differences in local mucosal immunity potentially explaining the varied clinical presentations [51]. Several studies have identified markers associated with malignancy in hyperplastic candidiasis lesions, including increased expression of p53, p21, and proliferating cell nuclear antigen, as well as elevated apoptosis rates [58].

Angular cheilitis is a multifactorial condition characterized by fissures and inflammation at the corners of the mouth. It is commonly associated with *Candida* species and *Staphylococcus aureus* infections [59,60]. Predisposing factors include denture wear, decreased vertical dimension of the face, and systemic conditions like anemia [59,61]. In denture wearers, replacing old dentures with new ones can significantly reduce *Candida* colonization and improve angular cheilitis [61].

Median rhomboid glossitis is a condition affecting the midline posterior tongue, occurring in approximately 0.7–1% of adults [62,63]. Once thought to be a developmental anomaly, current evidence suggests median rhomboid glossitis is primarily caused by *Candida* species [63]. The condition presents as an erythematous, smooth area lacking papillae and is characterized by pseudoepitheliomatous hyperplasia of the squamous epithelium. *Candida* infection induces proliferating acanthosis and superficial micro-pustules. While *Candida* is the primary cause, other microorganisms like Actinomyces have been implicated in some cases [64].

Denture stomatitis is a common inflammatory condition affecting denture wearers, with a global prevalence of 20–67% [65]. It is primarily associated with *Candida* species due to its virulence and ability to form biofilms on oral tissues and denture surfaces [66]. The etiology of denture stomatitis is multifactorial, involving factors such as ill-fitting dentures, continuous denture wear, poor oral hygiene, and compromised host immunity [65,67]. Diabetic patients are at higher risk for denture stomatitis compared to non-diabetics [68]. The presence of dentures can alter the oral microbiome and immune response, particularly in older individuals [67]. Management strategies include improving denture fit, enhancing oral hygiene, and addressing underlying systemic conditions [66].

## 4. Saliva

Saliva plays a crucial role in oral health as the first line of defense against colonization of microbial pathogens, including *Candida* species. Saliva contains various antimicrobial components that can inhibit the growth and adhesion of *Candida* species, and thus, reduced salivary flow, i.e., xerostomia, can lead to an overgrowth of *C. albicans* in the oral cavity. However, some proteins in saliva may promote adhesion of *Candida* species to oral surfaces.

### 4.1. Basic Proline-Rich Proteins (bPRPs)

bPRPs act as receptors for *C. albicans*, and specific bPRPs have been identified as binding sites [69]. These proteins are major components of the salivary pellicle and selectively adsorb to oral streptococci and hydroxyapatite surfaces. This process promotes *C. albicans* adhesion and enhances its colonization or that by mixed-species communities in the oral cavity [69]. *Candida* species can utilize proline as a sole energy source to enhance their virulence, as the proline catabolism pathway is essential for morphological switching critical for the transition from commensal to pathogenic states [70]. In addition, oral streptococci upregulate gene expression of *C. albicans* adhesins and enhance tissue invasion [6].

*C. albicans* surface mannoproteins, such as Bgl2p, a 35-kDa protein, have been identified as adhesins that can bind to immobilized salivary components, including bPRPs [71]. Bgl2p is a β-1,3-glucosyltransferase found in the cell wall of *Candida* species and contributes to cell wall assembly, biofilm formation, and adhesion to saliva-coated hydroxyapatite [72]. Bgl2p is involved in the transition from yeast to filamentous cells during biofilm development. Disruption of the BGL2 gene leads to attenuated virulence, increased sensitivity to chitin synthesis inhibitors, and slower growth rates [72,73]. Bgl2p expression increases in response to antifungal drug treatment, suggesting its involvement in antifungal resistance mechanisms [73].

### 4.2. Mucin

Mucins are glycoproteins that form a protective gel-like layer in saliva [74]. Mucins can block the adherence of certain microorganisms, including *C. albicans*, to oral surfaces by binding and aggregating with the fungal cells, thus preventing their attachment [75]. In addition, mucins suppress hyphal growth of *C. albicans* [75,76]. Mucins induce a unique oval-shaped morphology in *C. albicans*, downregulating genes related to adhesion and filamentation [75]. Recent research has identified specific mucin O-glycans as natural inhibitors of *C. albicans* pathogenicity, with core 1, core 1 + fucose, and core 2 + galactose structures showing potent anti-filamentation effects [77]. The binding of *Candida* species to mucins involves hydrophobic interactions and is concentration- and time-dependent. Different *Candida* species exhibit varying levels of adherence to small intestinal mucin, correlating with their virulence hierarchy [74]. However, *C. albicans* can enzymatically degrade mucins by secretory aspartyl proteinases (Saps) and potentially influence *Candida* populations in the oral cavity and gastrointestinal tract [74].

### 4.3. Histatin/Statherin

Histatins are a family of histidine-rich peptides secreted in human saliva with potent antifungal properties, particularly against *Candida* species [78,79], causing small membrane defects and nucleotide leakage [80]. These small, cationic peptides exhibit varying degrees of effectiveness, with histatins 1, 3, and 5 being the most studied [8,78,79,80,81,82,83,84,85,86]. A negative correlation between salivary histatin levels and oral yeast carriage has been reported, suggesting a role for histatins in maintaining oral health [4,87]. The antifungal mechanism of histatins differs from that of azole-based drugs and may involve endocytosis and endosomal disruption [88]. Synthetic histidine-rich peptides, especially those with higher degrees of branching, demonstrate enhanced antifungal activity compared with natural histatins [88]. Due to their broad-spectrum activity, low toxicity, and effectiveness against drug-resistant strains, histatins hold promise as therapeutic agents against fungal infections [86].

Statherin is a small, acidic salivary phosphoprotein composed of 43 amino acids (encoded by the STATH gene) that helps maintain the integrity of the salivary pellicle. *C. albicans* strains can bind to statherin [89], which in turn mediates fungal adhesion to hydroxyapatite and epithelial cells. Different strains show varying binding affinities [89]. Interestingly, statherin can also induce the transition of *C. albicans* from hyphae to yeast, potentially contributing to oral defense against candidiasis [85]. The C-terminal region of statherin is particularly important for binding to *C. albicans* [90]. In addition, statherin-derived peptides can reduce *C. albicans* biofilm formation and viability [91]. These findings highlight the complex relationship between statherin and *Candida* species in the oral cavity.

### 4.4. β-Defensin

Human β-defensins (HBDs) are produced by oral epithelial cells and salivary glands [92,93]. HBDs exhibit potent antifungal activity against *Candida* species through various mechanisms. HBD-1, HBD-2, and HBD-3 demonstrate fungicidal effects at micromolar concentrations, with HBD-2 and HBD-3 being more effective than HBD-1 [94,95]. They act in an energy-dependent and salt-sensitive manner, without causing significant membrane disruption [93]. HBD-2 specifically targets phosphatidylinositol 4,5-bisphosphate (PIP2) in the fungal cell membrane and leads to cell permeabilization and death [96]. In addition, HBDs can inhibit *Candida* adherence to oral epithelial cells, thereby regulating their own expression in response to *C. albicans* hyphae [95]. The candidacidal effects of HBDs can be additive or complementary when combined with other antimicrobial peptides like histatin 5 [93]. While HBD-1 expression is constitutive, HBD-2 expression can be induced by inflammatory stimuli such as IL-1β and bacterial lipopolysaccharide [92]. Salivary defensin levels may be altered in various oral diseases, such as oral lichen planus, leukoplakia, and glossitis associated with iron deficiency [97,98,99].

α-defensins are primarily derived from neutrophils and are found in high concentrations in saliva (1–10 μg/mL, effective concentration against *Candida* species), as they are present in significant quantities in gingival crevicular fluid [100,101]. Therefore, totally edentulous patients show significantly lower levels of α-defensins compared with non-edentulous individuals, likely due to the absence of gingival sulcus [102]. Elevated levels of human neutrophil peptide-1, i.e., α-defensin-1, have been observed in the saliva of patients with oral squamous cell carcinoma, showing a positive correlation with serum squamous cell carcinoma antigen levels [103]. In cases of oral candidiasis, both α-defensins and HBD-2 show increased expression in the buccal epithelium than in normal tissue [104]. However, two-fold and four-fold higher amounts of α-defensins were observed in patients with aggressive and chronic periodontitis, respectively, compared with healthy controls [105].

### 4.5. Secretory Immunoglobulin A

Secretory immunoglobulin A (sIgA) plays a role in immune defense in the oral cavity. It binds to *Candida* species, potentially preventing adhesion to oral surfaces by neutralizing their ability to interact with host tissues [106,107]. Recent research demonstrates that sIgA inhibits *C. albicans* hyphal growth and virulence by reducing the levels of ergosterol, a key component in fungal cell membranes [107]. In animal models, increased levels of specific sIgA antibodies correlate with decreased intestinal *Candida* colonization. Moreover, women with vulvovaginal candidiasis exhibit lower salivary sIgA levels compared with healthy controls, suggesting a protective role of sIgA against *Candida* infections [108].

### 4.6. Other Salivary Proteins

Lactoferrin, an antimicrobial protein, is also fungicidal to some *Candida* species, including *C. albicans*, *C. tropicalis*, *C. parapsilosis*, and *C. dubliniensis*, with varying susceptibility among and within species [109,110]. The antifungal mechanism of lactoferrin involves both iron deprivation and direct interaction with fungal cell surfaces, causing cell wall changes [109]. Lactoferrin has demonstrated synergistic effects with azole antifungals, particularly against resistant *Candida* strains, possibly owing to its iron-chelating function [111].

The antimicrobial peptide LL-37 exhibits significant antifungal activity against *Candida* species [80]. At sub-lethal concentrations, LL-37 inhibits *C. albicans* adhesion to various surfaces by binding to cell wall carbohydrates, particularly mannan. LL-37 and its analogs exert potent antifungal effects, with some showing minimum inhibitory concentrations as low as 0.07 µM against *C. albicans* and *C. tropicalis* [112]. The mechanism of action of LL-37 involves severe disruption of the fungal cell membrane, causing disintegration into discrete vesicles, and the efflux of both small and large molecules [80].

Binding interactions between *Candida* species and human salivary molecules are summarized in Table 2.

The balance of proteins and their interactions with *Candida* species and other oral microorganisms are critical for maintaining oral health. Disruptions in this balance due to salivary gland dysfunction, changes in diet, or other causes can increase the risk of oral diseases, including candidiasis.

Amylase breaks down starches into sugars, which can serve as nutrients for *C. albicans*, potentially enhancing its growth and adhesion in the oral cavity. *Candida* species can produce extracellular amylases [113], with various influencing factors including pH, carbon sources, and nitrogen sources. Metabolic differences exist among *Candida* species; some species, such as *C. albicans* and *C. auris*, show enhanced amino acid metabolism that may contribute to their pathogenicity [113].

## 5. Virulence Factors of *Candida* Species

### 5.1. Secretory Aspartyl Proteinases (Saps)

Saps are critical *Candida* virulence factors that contribute to pathogenicity through adhesion, invasion, and tissue damage [114,115]. The proteolytic activity of Saps allows *Candida* species to escape the first line of host defense, as described above. The SAP gene family, consisting of 10 genes, is differentially distributed among *Candida* species, with non-pathogenic species typically having fewer SAP genes [115,116]. Sap expression varies depending on environmental conditions, morphology, infection stage, and host response [6]. Different Saps may play distinct roles in virulence, as evidenced by their varying primary sequences and pI values [86,114,116]. Exposure to subinhibitory concentrations of antifungal agents, such as azoles, can also upregulate Sap expression in resistant strains [117].

*Candida* species have developed mechanisms to evade host immune systems, including the complement system. Saps contribute to this evasion process; Sap1, Sap2, and Sap3 degrade complement components C3b, C4b, and C5 and inhibit the formation of the terminal complement complex [114]. The absence of terminal complement factors reduces phagocytosis of *Candida* species by polymorphonuclear leukocytes [118]. Saps, especially Sap2 and Sap6, induce neutrophil chemotaxis both in vitro and in vivo [116,119], and stimulate the production of chemokines, such as IL-8, by oral and vaginal epithelial cells, contributing to aseptic inflammation [119,120].

Saps not only degrade proteins but also participate in biofilm formation. Sap9, a cell wall-associated proteinase, is involved in fungal cell–cell recognition and interkingdom communication during polymicrobial biofilm development [121]. Exposure to subinhibitory concentrations of fluconazole augments Sap production in *C. albicans* biofilms, with higher secretion observed at lower cell densities [122].

Inhibition of Sap activity has been proposed as a potential treatment strategy for *Candida*-associated infections. For instance, mycogenic silver nanoparticles have significant inhibitory effects on biofilm growth and Sap activity in both *C. albicans* and non-*albicans Candida* species [123].

### 5.2. Candidalysin

Candidalysin, first identified in *C. albicans*, is a fungal cytolytic peptide toxin critical for mucosal and systemic infections [124]. The 31-amino acid peptide plays a crucial role in the virulence of *Candida* species, particularly in mucosal infections, as it damages epithelial membranes, triggers a danger response signaling pathway, and activates epithelial immunity [124,125]. Candidalysin is unique among fungal toxins in that it has the ability to directly damage various host cells and activate immune responses [126,127] via epidermal growth factor receptor (EGFR) activation, which involves matrix metalloproteinases, EGFR ligands, and calcium [128]. Candidalysin is also essential for neutrophil recruitment and virulence during systemic *C. albicans* infections because it can activate MAPK signaling and induce chemokine secretion by endothelial cells [129]. Recently, candidalysin orthologs were discovered in *C. dubliniensis* and *C. tropicalis*, forming a new family of fungal peptide toxins [130,131]. The orthologs have different amino acid sequences but are all amphipathic and predominantly α-helical in structure. Variations in the amino acid sequence influence the toxicity and biological activity of candidalysin. Notably, the damaging and activation potential of candidalysin in *C. dubliniensis* and *C. tropicalis* is higher than that in *C. albicans*, exhibiting more rapid membrane binding and disruption [130]. Candidalysin—which induces the release of proinflammatory cytokines including IL-17, recruits neutrophils and other immune cells to the site of infection and amplifies host immune responses [131,132]—is a key driver of host cell activation and Th17 immunity essential for combating fungal infections [2,127]. In addition, candidalysin induces platelet release from oral megakaryocytes [2]. The presence of candidalysin is associated with various conditions, including oropharyngeal candidiasis and vulvovaginal candidiasis, where it contributes to the inflammatory response observed in infections with *Candida* species.

Candidalysin is derived from the larger protein Ece1 (extent of cell elongation 1) through proteolytic processing by Kex2 and Kex1 proteases [133]. The mechanism of action of candidalysin involves not only direct membrane damage but also activation of the nucleotide-binding oligomerization domain-like receptor family pyrin domain-containing 3 (NLRP3) inflammasome in macrophages, which is crucial for the inflammatory response during infections [126,129]. The activation of the NLRP3 inflammasome leads to the release of IL-1β, a key cytokine in the immune response. Moreover, candidalysin also has a direct inhibitory effect on certain bacterial species, suggesting its role in shaping the microbial environment during infections [125]. This dual action of damaging host cells while also influencing bacterial populations reflects the complexity of the role of candidalysin in the pathogenicity of *Candida* species.

*Nakaseomyces glabratus*, formerly known as *Candida glabrata*, is also a significant opportunistic human fungal pathogen that causes invasive infections, although it is unable to form hyphae and has no candidalysin ortholog [5,9]. *N. glabratus* is the third or fourth most common *Candida* species responsible for candidemia and invasive candidiasis, with an estimated 1.5 million annual cases reported globally [134]. *N. glabratus* exhibits increased resistance to azole antifungals and echinocandins, complicating treatment options, and due to the growing threat it poses, the World Health Organization has designated *N. glabratus* as a high-priority fungal pathogen [5,9]. While the name change (from *Candida glabrata* to *Nakaseomyces glabratus)* has been adopted by some researchers, others argue that it may hinder clinical practice and public health efforts [134]. Despite the challenges, amphotericin B remains a viable treatment option for *N. glabratus* infections [9].

### 5.3. Agglutinin-Like Sequence (Als) Proteins

**Figure 2:**
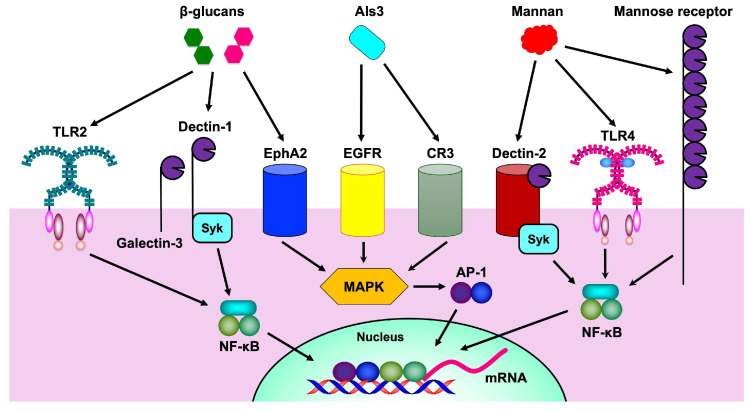
Receptors for components of *Candida* species. NF-κB and AP-1 are transcription factors, which play an important role in cytokine production. Abbreviations: Als, agglutinin-like sequence; EGFR, epidermal growth factor receptor; EphA2, ephrin type-A receptor 2; CR3, complement receptor 3; TLR, Toll-like receptor; Syk, spleen tyrosine kinase; NF-κB, nuclear factor-κB; MAPK, mitogen-activated protein kinase; AP-1, activator protein-1.

In *Candida* species, adhesion to host cells, invasion, and biofilm formation require Als proteins. The N-terminal domain of Als proteins is responsible for substrate-specific binding, with the N-terminal domain of Als1 protein showing affinity for fucose-containing glycans and extracellular matrix proteins, such as type IV collagen, fibronectin, and laminin [135,136]. Fibronectin facilitates fungal adherence to host surfaces but also acts as an opsonin in the bloodstream, enhancing phagocytosis [136]. Structural analysis revealed that Als adhesins bind to flexible C-termini of host cell proteins through a conserved lysine residue and a network of water molecules, allowing recognition of diverse sequences [137]. Eight Als proteins, Als1–7 and Als9, have been identified in *C. albicans* [135,138]. Als3 is particularly important for epithelial adhesion, cell damage, and cytokine induction (Figure 2) and functions as both an adhesin and invasin to mediate the attachment of *Candida* species to the salivary pellicle, host cells, and extracellular matrix proteins, inducing host cell endocytosis and enabling iron acquisition from host ferritin, a major iron storage protein [136,138,139,140]. However, Als proteins also act as CR3 ligands to promote inflammasome activation in anti-*Candida* species immunity [141]. In addition, Als3 binds to caspase-8 and apoptosis-associated speck-like protein containing a caspase recruitment domain (ASC), activating NLRP3 inflammasome in the cytosol [142]. The Als family is present in other *Candida* species as well, with five ALS genes characterized in *C. parapsilosis*, four in *C. metapsilosis*, and three in *C. orthopsilosis* [143]. They share similarities with the *C. albicans* Als family but also have unique properties, which blur the boundaries among fungal cell wall protein families [143].

The relationship between EGFR and Als proteins of *Candida* species is important in the pathogenesis of oropharyngeal candidiasis. EGFR and human epidermal growth factor receptor 2 (HER2) interact with *C. albicans* in an Als3-dependent manner, inducing receptor autophosphorylation and epithelial cell endocytosis [144]. Als3 potentiates the targeting of the pore-forming toxin candidalysin, which activates EGFR signaling [132]. EGFR also forms a complex with ephrin type-A receptor 2 (EphA2), and both are mutually dependent on *C. albicans*-induced activation [132]. Als3 and candidalysin interact with this complex, leading to epithelial cell damage and sustained inflammatory responses [132].

These interactions suggest the complex interplay between virulence factors of *Candida* species and host cell receptors in cancer, with EGFR, HER2, and EphA2 being receptor tyrosine kinases implicated in cancer progression [132,144].

### 5.4. Ssa1/Ssa2

Heat shock proteins are required for resistance to host-derived stress, such as antimicrobial peptides [145]. Ssa1, a heat shock protein in *C. albicans*, functions as a cell surface receptor for antimicrobial peptides such as histatins and acts as an invasin that binds to host cell cadherins and EGFR/HER2, inducing endocytosis [144,145]. Ssa1 is abundant in the hypoxic secretome of *C. albicans* and exerts immunomodulatory effects on macrophages, potentially inhibiting *C. albicans* uptake [146]. Targeting Ssa1 with monoclonal antibodies has shown promise in preventing *C. albicans* adhesion to and invasion of host cells, offering a potential new treatment strategy for invasive fungal infections [147].

Ssa2, another heat shock protein in *Candida* species, plays a crucial role in the binding and intracellular transport of histatin 5 [83,84]. While both Ssa1 and Ssa2 in yeast cells serve as binding receptors for histatin 5 (i.e., deletion of these proteins hinders histatin 5 binding) [83], Ssa2 has a higher affinity and is more important for the fungicidal activity of histatin 5 [83]. The ATPase domain of Ssa2 contains specific binding sites for histatin 5, and mutations in these sites reduce the intracellular transport and antifungal effects of histatin 5 [84]. Unlike Ssa1, however, Ssa2 is not critical for *C. albicans* virulence in murine models of disseminated and oropharyngeal candidiasis [145].

### 5.5. Other Adhesins

Hyphal wall protein 1 (Hwp1), an adhesin expressed on the surface of *Candida* hyphae, takes part in biofilm formation and host cell adhesion via fibronectin [136,139,148]. Similarly, Hwp2 plays an essential role in the adhesion, biofilm formation, and oxidative stress tolerance of *Candida* species [149]. In *C. albicans*, Hwp1 functions as a substrate for mammalian transglutaminases, forming covalent cross-links with epithelial cells [150]. In vivo studies have demonstrated that Hwp1 is necessary for biofilm formation in catheter models and contributes to virulence in oroesophageal candidiasis [148,150]. The *HWP1* gene has been identified as a potential marker for differentiating between different species of *Candida*, including *C. tropicalis*, *C. parapsilosis*, *C. orthopsilosis*, and *N. glabratus*. Phylogenetic analysis based on the *HWP1* gene showed consistency with other gene-based analyses, suggesting that it may serve as an excellent marker not only for the identification but also in phylogenetic studies of clinically significant *Candida* species [151].

Bcr1 is a transcription factor upstream of Als3, Hwp1, and Ece1 in *Candida* species [152]. During the development of oropharyngeal candidiasis, Bcr1 influences the expression of *ALS3*, although its impact on gene expression is more limited in vivo relative to in vitro conditions [153]. Bcr1 also regulates the expression of common fungal extracellular membrane (CFEM) proteins, which are involved in iron acquisition from heme and biofilm formation by *C. albicans* [152,154]. In *C. parapsilosis*, Bcr1 regulates CFEM proteins for iron acquisition but not for biofilm formation [154]. However, the importance of Bcr1 in biofilm formation was shown to vary among clinical isolates of *C. parapsilosis*, with some strains showing Bcr1-independent biofilm formation [155].

CR3-related protein (CR3-RP) is a surface antigen expressed by *Candida* species and is structurally and functionally similar to human CR3 [156]. CR3-RP is a key surface antigen expressed by *C. auris*, the emerging pathogen, and is involved in the adherence and the initial phase of biofilm formation [157]. Anti-CR3-RP antibodies have shown potential in inhibiting *C. auris* biofilm formation and eradicating pre-formed biofilms, demonstrating efficacy comparable to that of conventional antifungals [157]. Similar effects have been observed in related species, such as *C. albicans* and *C. dubliniensis*, in which anti-CR3-RP antibodies reduced fungal adherence and biofilm thickness [158]. The antibodies also demonstrated efficacy in ex vivo and in vivo models, suggesting their potential for use in immunotherapy or vaccine development against biofilm-associated *Candida* infections [158]. CR3-RP has been identified as a promising marker for *Candida* infections, with synthetic CR3-RP eliciting significant IgM and IgA antibody responses in patients with recurrent vulvovaginal candidiasis [156].

pH-regulated antigen 1 (Pra1), similar to CR3-RP, is a surface-associated and secreted protein produced by *Candida* species, particularly in its hyphal form [1,159]. In *C. albicans*, Pra1 plays a complex role in fungal recognition and immune response. Pra1 can enhance neutrophil migration and adherence when surface-bound but inhibits neutrophil activation when secreted [159]. Pra1 is also a potent complement inhibitor, blocking C3 activation and reducing complement-mediated adhesion and uptake by macrophages [160]. Pra1 cleaves C3 at a unique site and inhibits the effector functions of its activation fragments, thereby disrupting host complement attack [160]. In addition, Pra1 directly binds to mouse CD4^+^ T cells, increasing their proliferation but reducing effector cytokine secretion upon stimulation [161]. As a ligand for CR3 (leukocyte integrin αMβ2), Pra1 mediates adhesive and migratory responses to *C. albicans* [159]. The PRA1 gene is maximally expressed at a neutral pH, and its deletion affects hypha formation [162]. These findings suggest that Pra1 plays a crucial role in the pathogenicity of *C. albicans* by modulating host immune responses and contributing to fungal morphogenesis. In *C. tropicalis*, Pra1 was found to inhibit complement activation in all three pathways and was expressed at higher levels in clinical isolates relative to reference strains [1]. Pra1 binds to complement components C3, C3b, factor-H, and C4BP, inhibiting complement activation and reducing C3b/iC3b surface deposition [1].

### 5.6. Mannan

Mannan, a key component of the *Candida* cell wall, plays a crucial role in oral health by mediating interactions with host cells and immune responses. Different *Candida* species exhibit unique mannan structures, influencing their pathogenicity and immune recognition [15,163]. *C. albicans* serotype A mannan determinants are particularly important for adherence to buccal epithelial cells, potentially affecting colonization and infection [164]. Mannan from various *Candida* species differentially activates dendritic cells, with *C. albicans* mannan potentially skewing T helper responses towards Th1, while *C. parapsilosis* mannan induces strong proinflammatory responses [165]. Interestingly, oral *Candida* mannan concentrations correlate with symptoms of ill health as well as immune status [166].

The mannose receptor, a C-type lectin receptor, recognizes mannan (Figure 2) and contributes to the phagocytosis of unopsonized *C. albicans* by macrophages [167,168]. The interaction between the mannose receptor and *C. albicans* decreases mannose receptor endocytic activity without immediate receptor degradation [168]. Interferon-γ enhances macrophage candidacidal activity, potentially involving the mannose receptor [167]. The unique mannan structure of each *Candida* species, particularly the β-1,2-linked mannose units, is highly antigenic and plays a role in adhesion to epithelial cells and pathogenicity [163].

TLR4 also plays a crucial role in the immune response against mannan of *Candida* species. TLR4 polymorphisms Asp299Gly and Thr399Ile are associated with increased susceptibility to *Candida* bloodstream infections and elevated IL-10 production [169]. TLR4 specifically binds to O-linked mannosyl residues on the *Candida* cell wall, and N-linked mannans are recognized by the mannose receptor [15]. This recognition is important for cytokine production by immune cells, with TLR4 and CD14 being essential for mannan-induced tumor necrosis factor alpha (TNF-α) production in human monocytes [170]. TLR4 is also responsible for the protective effect of a *C. albicans* extracellular vesicle-based vaccine prototype in murine systemic candidiasis [171]. Additionally, TLR4 expression on hematopoietic stem and progenitor cells may modulate hematopoiesis upon *C. albicans* recognition, offering potential avenues for anti-*Candida* immunointervention [172].

Dectin-2, a C-type lectin receptor expressed on dendritic cells and macrophages, recognizes α-mannans in fungal cell walls [7]. Upon ligand binding, dectin-2 activates the spleen tyrosine kinase (Syk)-caspase recruitment domain protein 9-nuclear factor (NF)-κB signaling pathway, inducing cytokine production and Th17 cell differentiation [7]. Dectin-2-deficient mice demonstrate an increased susceptibility to systemic candidiasis, which is correlated with higher fungal loads and decreased cytokine production [173]. The importance of dectin-2 varies among *Candida* species exhibiting differing mannan content in their cell walls [163,174]. Dectin-2 is crucial for the clearance of *C. albicans*, *C. tropicalis*, *C. parapsilosis*, and *N. glabratus* during systemic infection, although its role in cytokine production differs among species [154].

These findings highlight the complex role of *Candida* mannan in oral health, influencing both host–pathogen interactions and immune responses.

### 5.7. β-Glucan

β-glucan, a cell wall polysaccharide of *Candida* species, plays a complex role in oral health. The *Candida* cell wall consists of an inner layer of chitin and β-glucan and an outer layer of mannan, although β-glucan was expressed at the surface of heat-killed *Candida* yeast [15,175]. β-glucan is exposed during mucosal biofilm growth and more uniformly present on the surface of biofilm organisms invading the oral mucosa [176]. Interestingly, β-glucanase produced by *Lactobacillus acidophilus* has shown promise in disrupting biofilm formation by *Candida* species, which is associated with denture stomatitis [177]. However, *Candida* β-glucan induces ROS production in human neutrophils to improve the killing of *C. albicans* and *N. glabratus* [178].

Dectin-1, a myeloid receptor, plays a crucial role in β-glucan recognition [179,180] which is essential for antifungal immunity. Dectin-1 deficiency leads to increased susceptibility to *C. albicans* infections in mice [179]. The interaction between dectin-1 and β-glucan triggers cytokine production in human and murine immune cells [179]. Notably, heat-killed *C. albicans* exposes more β-glucan at the surface than live *C. albicans*, enhancing dectin-1 recognition, while live yeast primarily stimulates monocytes through mannans [175,179]. Dectin-1 induces cytokine production via two pathways: Syk-dependent anti-inflammatory IL-10 production and TLR-dependent proinflammatory cytokine stimulation [179]. However, some immune responses, such as Th1-type cytokine production, occur independently of dectin-1 [179]. Dectin-1 also activates CR3, namely Mac-1, in neutrophils to increase phagocytosis, although *Candida* species can also bind to CR3 via Als proteins and Pra1 [181].

TLR2 plays a crucial role in the recognition of β-glucan in the cell wall of *C. albicans*, often in collaboration with dectin-1 [15]. While TLR2 is important for cytokine production in response to *C. albicans*, its role is complex and can be both pro- and anti-inflammatory. TLR2 recognition of *C. albicans* can induce proinflammatory cytokines but also promotes immunosuppression through increased IL-10 production and regulatory T cell survival [182]. This immunosuppressive effect may explain why TLR2-deficient mice show increased resistance to disseminated candidiasis [182].

EphA2 functions as a pattern recognition receptor in oral epithelial cells, binding not only to Als3 but also to β-glucan in *C. albicans*, and triggers proinflammatory responses [183]. EphA2 plays an important role in the host defense against *C. albicans* during the development of oropharyngeal candidiasis. Indeed, EphA2 knockout mice exhibit lower IL-17 production compared with wild-type mice [184]. EphA2 is also expressed on neutrophils and enhances antifungal activity by augmenting ROS production [184].

These interactions highlight the complex interplay between virulence factors of *Candida* species and host cell receptors in oral inflammatory responses and tumor progression. Molecules of *Candida* species and molecular interactions in humans are summarized in Table 3.

## 6. *Candida* Species and Oral Diseases

### 6.1. Candida Species and Dental Caries

*Candida* species, particularly *C. albicans*, are common oral fungal pathogens associated with various oral health problems. Poor oral hygiene is significantly associated with increased *Candida* colonization [185]. Patients with xerostomia have higher *Candida* counts, with specific *Candida* species linked to particular oral sites [186], and more oral mucosal disorders compared with healthy individuals. Recent studies suggest a potential relationship between *Candida* species and dental caries [187,188,189]. In particular, *C. albicans* is frequently found in the oral cavity and has cariogenic characteristics, such as adhesion to dental tissues, acidogenicity, and collagenolytic enzyme production [139,188,190]. Patients with caries show higher *Candida* colonization rates and species diversity than those in caries-free individuals [188,189]. Some studies proposed the use of *C. albicans* as a caries risk indicator, while others suggested that it functions primarily as a secondary agent, particularly in the development of dentinal caries [191]. The interaction between *C. albicans* and other oral bacteria, particularly *Streptococcus mutans*, may contribute to caries development [187]. *C. albicans* hyphal formation is suppressed in the presence of *S. mutans*, while *S. mutans* adherence and mixed biofilm formation are enhanced by *C. albicans* both in vitro and in vivo [192,193,194,195]. Dual-species biofilms of *C. albicans* and *S. mutans* exhibit greater biomass, structural complexity, and stress tolerance compared with single-species biofilms [196]. Transcriptomic analysis revealed altered gene expression in dual-species biofilms, with upregulation of genes related to microbial metabolism, acid adaptation, and oxidative stress response [197]. While Als1, Als3, and Hwp1 are important for *C. albicans* single-species biofilm formation, Als1 and Hwp1, but not Als3, are major players in dual-species biofilm formation by *C. albicans* and *S. mutans* [198]. Glucosyltransferase B (GtfB), secreted by *S. mutans*, adheres to *C. albicans* cell surfaces in an enzymatically active form, produces glucans that enhance interactions between the two species as well as with tooth enamel [187,190], and binds to *Candida* mannan [199]. *S. mutans* antigen I/II is required for two-species biofilm formation [200]. A recent study demonstrated that, although *S. mutans* can adhere to *C. albicans* and use its mycelial structures to traverse surfaces, the bacterium is unable to move or spread independently [201]. The cross-kingdom interactions impact biofilm formation dynamics and virulence gene expression, potentially contributing to the development of dental caries and oral candidiasis.

While *C. albicans* remains the most prevalent oral yeast, non-*albicans Candida* species show significantly higher colonization in patients with caries than in those without [202,203]. The severity of caries in preschool children is correlated with increased *Candida* carriage rates and diversity, including rare non-*albicans* species like *C. dubliniensis* [202]. Non-*albicans Candida* species isolated from dental caries lesions exhibit virulence factors comparable to those of *C. albicans*, including biofilm production, phospholipase activity, and hemolysis [203]. This suggests that non-*albicans Candida* species may also contribute to caries formation. The cariogenic potential of *Candida* species is attributed to their adhesion properties, acidogenicity, acid tolerance, and ability to produce collagenolytic proteinases.

The *Lactobacillaceae* family has shown significant potential in inhibiting *Candida* colonization and growth at various human body sites, including the oral cavity. Bacteria of this family can interfere with the growth of *Candida* species through multiple mechanisms, including the production of acetic acid, lowering pH, and secreting metabolites that affect fungal growth and virulence [177,204]. They can also enhance host defense mechanisms against *Candida* species [205]. However, *C. albicans* and *N. glabratus* were found to establish close contacts with *L. gasseri* and *L. crispatus* and form mixed biofilms, at least in vitro [204,206].

### 6.2. Candida Species and Periodontitis

There is no significant difference in *Candida* prevalence between patients with and without chronic periodontitis [207]. *C. albicans* protects *Porphyromonas gingivalis*, a periodontal pathogenic anaerobe, from an unfavorable aerobic environment [208,209]. In addition, *C. albicans* and *P. gingivalis* can coadhere through specific proteins, notably InlJ and Als3 [208,210]. The presence of *C. albicans* induces gingipain activity in *P. gingivalis*, increasing its virulence [209]. Moreover, *C. albicans* enhances *P. gingivalis* invasion of human gingival epithelial cells and fibroblasts, potentially exacerbating periodontal disease [211]. These interactions demonstrate the significance of polymicrobial communities in oral infections and highlight the potential role of *C. albicans* in promoting *P. gingivalis* pathogenicity.

The relationship between *Candida* species and *Fusobacterium nucleatum,* a periodontal pathogen, involves complex interactions that can influence their virulence and colonization in the oral cavity. *C. albicans* and *F. nucleatum* coaggregate through specific cell surface components, including the *C. albicans* adhesin Flo9 and the *F. nucleatum* adhesin RadD [212]. These interactions inhibit hyphal morphogenesis and growth of *C. albicans* and potentially promote a commensal lifestyle in the oral cavity [213]. Moreover, coaggregation of *F. nucleatum* and *Candida* species is mediated by a heat-labile component on the surface of *F. nucleatum* and a mannan-containing heat-stable receptor on *Candida* species [214]. Interestingly, *C. dubliniensis* also coaggregates with *F. nucleatum* at 37 °C, while its coaggregation with *C. albicans* requires different growth conditions or treatments [214].

### 6.3. Other

Several studies have investigated the relationship between peri-implantitis and *Candida* species. Some suggested a higher prevalence of *Candida* in peri-implantitis, while others found no significant difference in the occurrence of *Candida* between peri-implantitis and healthy implants [215,216]. Dentate individuals and those with peri-implantitis have an increased presence of *Candida* (3–76.7%), with *C. albicans* being consistently reported as the most common species isolated, followed by *C. parapsilosis*, *C. tropicalis*, and *C. dubliniensis* [215,217]. The peri-implant sulcus serves as a suitable niche for colonization by *Candida* species [216]. Factors such as total edentulism and the use of implant-fixed complete prostheses or implant-retained removable prostheses may influence *Candida* presence [216]. Further studies are warranted to establish the role of *Candida* species in peri-implantitis and their interactions with other microorganisms. Understanding these relationships, as well as the molecular aspects of *Candida* behavior, is essential for developing effective preventive and therapeutic measures for oral candidiasis.

The interactions between *C. albicans* and oral microorganisms are summarized in Table 4. Their interactions play crucial roles in oral colonization and polymicrobial pathogenesis and may offer potential targets for new treatment strategies.

## 7. *Candida* Species and Cancer

Recent studies suggest a significant relationship between *Candida* species and cancer development. *Candida* infections are associated with increased short- and long-term cancer risks [218]. *Candida* species have been linked to several types of cancer, including oral, esophageal, gastric, pancreatic, colorectal, liver, breast, and skin cancers [219,220]. *C. albicans* may promote cancer through various mechanisms, including the production of carcinogenic byproducts and induction of inflammation [219,220]. Moreover, *C. albicans* may contribute to cancer development via additional mechanisms that involve modulation of the immune system, induction of matrix metalloproteinases, overexpression of metastasis-related genes, damage to the mucosal epithelium, alteration of the microbiome, activation of oncogenic signaling pathways, and production of carcinogenic metabolites such as nitrosamine and acetaldehyde.

Recent studies suggest a significant association between *Candida* species and oral squamous cell carcinoma (OSCC). A systematic review and meta-analysis found a higher frequency of *Candida* species in OSCC patients compared to healthy individuals [221,222]. In addition, *Candida* spp. and *Saccharomyces* spp. were also detected in gastrointestinal cancer samples, and the ratio (C/S) was significantly increased in stage IV [219]. *Candida* spp. are predictors of decreased survival in patients with head and neck cancer and colorectal cancer [219]. *C. albicans* has been linked to cancer development through various mechanisms, including the production of carcinogenic byproducts and triggering inflammation [222]. A case-control study reported a 70% prevalence of oral candidal carriage in OSCC patients compared to 20% in healthy cohorts, with both *C. albicans* and non-*albicans Candida* equally distributed [223]. Another meta-analysis confirmed an association between total *Candida*, *C. albicans*, and OSCC, although the evidence for non-*albicans Candida* species was weaker [224].

Research also indicates a significant association between oral potentially malignant disorders (OPMDs) and *Candida* species. Studies have found a higher prevalence of oral candidal carriage in patients with OPMDs compared to healthy individuals [225,226,227]. *C. albicans* is commonly isolated, but non-*albicans* species like *C. tropicalis* and *N. glabratus* are also prevalent [227,228]. The presence of *Candida* species is particularly associated with leukoplakia and oral submucous fibrosis [227]. *Candida* may act as an opportunistic pathogen, potentially contributing to cancer development in OPMDs [229]. Notably, patients with OSCC show even higher candidal carriage rates than those with OPMDs [228]. The strong association between *Candida* and OPMDs suggests that these fungi could be a risk factor for the progression of OPMDs to OSCC [225].

*Candida* species rapidly upregulate the secretion of galectin-3, a β-galactoside-binding C-type lectin expressed on various cells [230]. Galectin-3 mediates cancer progression and metastasis, as it is involved in various cancer cell activities, including growth, transformation, apoptosis, angiogenesis, adhesion, invasion, and metastasis [231,232,233]. Galectin-3 expression is modulated in cancer cells, making it a potential diagnostic/prognostic marker for specific cancer types [232]. Notably, levels of circulating galectin-3 are increased in cancer patients, promoting metastasis through its interaction with cancer-associated mucin 1; this causes mucin 1 polarization and exposure of smaller cell surface adhesion molecules. The interaction enhances cancer cell adhesion to endothelial cells, increases transendothelial invasion, and decreases the latency of experimental metastasis [232]. However, galectin-3 binds to and kills *Candida* species [234,235]. These findings emphasize the importance of controlling *Candida* infections during cancer treatment and exploring new therapeutic approaches to mitigate their pro-tumor effects.

β-glucan, on the other hand, demonstrates significant antitumor effects and could potentially inhibit tumor growth by enhancing the efficacy of antitumor monoclonal antibodies via activation of complement and priming neutrophils to kill opsonized tumor cells [236]. β-glucan modulates the tumor microenvironment by promoting M1-like polarization of tumor-associated macrophages and inducing ferroptosis in lung cancer cells [237,238]. The particulate form of β-glucan activates dendritic cells and macrophages via dectin-1, thereby stimulating potent antitumor immune responses and suppressing immunosuppressive cells [239]. However, soluble β-glucan enhances the efficacy of antitumor monoclonal antibodies through complement activation, independent of dectin-1 [239]. In addition, treatment with β-glucan was shown to enhance the sensitivity of lung cancer cells to etoposide, a cytotoxic drug for lung cancer [240].

## 8. *Candida* Species and Virus Diseases

*Candida* biofilms encompass viruses such as herpes simplex virus (HSV) and coxsackievirus type B5 and provide protection against antiviral drugs and the immune system, potentially serving as reservoirs for viral pathogens [241]. In addition to inducing prostaglandin E2 (PGE2) production in host cells, *C. albicans* can itself produce PGE2 [242]. PGE2 inhibits the production of type I interferons that are crucial for antiviral immunity and aids viral protection and replication [243]. However, HSV infection of host cells significantly enhances *C. albicans* adherence; both HSV-1 and HSV-2 increase yeast and germ tube form attachment by approximately two-fold and promote biofilm formation [244]. On the other hand, human immunodeficiency virus (HIV)-1 gp41, but not gp120, binds to *C. albicans* via complement C3-like regions and potentially augments oral candidiasis in HIV-infected individuals [245]. HIV-1 Tat protein also binds to *C. albicans* and increases phagocytosis of opsonized *C. albicans*, although Tat accelerates hyphal growth [246].

Recent studies revealed a significant relationship between SARS-CoV-2 infection and histatin production in salivary glands: SARS-CoV-2 infection led to decreased expression of histatin genes and reduced histatin-5 levels in saliva [4]. This reduction in histatin-5 is associated with an increased prevalence of *C. albicans* in patients with COVID-19 and appears to be reversible in mild-to-moderate cases, with levels tending to increase during the post-acute phase [4]. These findings suggest that SARS-CoV-2 infection may compromise oral innate immunity and predispose patients to oral candidiasis [4,247]. Long-term effects on oral health post-COVID-19 have also been observed, including chronic oral dysesthesia and dysgeusia [247].

SARS-CoV-2 infection suppresses immunity, increasing the susceptibility of patients to co-infections with fungi, including *C. auris* [248], an emerging multidrug-resistant fungal pathogen. *C. auris* has a remarkable ability to form dry surface biofilms that contribute to its persistence in healthcare environments. Dry surface biofilms enable *C. auris* to tolerate adverse conditions, including desiccation and sodium hypochlorite disinfection [46]. Compared with *C. albicans*, *C. auris* exhibits enhanced biofilm formation in synthetic sweat media, mimicking skin niche conditions [249]. This propensity to form biofilms on the skin may explain the efficient colonization and transmission behaviors of *C. auris* in healthcare settings. The ability to form robust biofilms in skin-like conditions, coupled with resistance to desiccation and disinfectants, likely contributes to the success of *C. auris* as a nosocomial pathogen and its capacity to cause outbreaks in healthcare facilities. Reportedly, *C. auris* was the causative agent in two-thirds of COVID-19 cases with candidemia in the intensive care unit (ICU) in New Delhi, with a mortality rate of 60% [248].

## 9. Conclusions

This review summarizes the role of saliva in regulating *Candida* growth and virulence. Saliva contains a wide range of antimicrobial proteins, including histatins, mucins, and defensins, which exert antifungal activity. However, certain salivary proteins, such as bPRPs and statherin, facilitate *Candida* adhesion, highlighting the dual nature of saliva in modulating fungal presence. The reduced salivary flow observed in conditions such as xerostomia may promote fungal overgrowth, increasing the risk of oral candidiasis.

The virulence mechanisms of *Candida* species have been extensively researched. The present review reaffirms the role of key virulence factors, including Saps, candidalysin, and Als proteins. These factors enable *Candida* species to adhere to host tissues, evade immune responses, and invade epithelial barriers. Notably, non-*albicans Candida* species exhibit distinct virulence traits; for example, *C. tropicalis* exhibits enhanced biofilm-forming ability, and *C. auris* demonstrates multidrug resistance, posing a significant public health concern. Thus, there is a need for species-specific treatment strategies, especially given the rising incidence of non-*albicans Candida* infections.

This review also explores the complex interactions between *Candida* species and bacterial cohabitants in the oral cavity. The synergistic relationship between *C. albicans* and *S. mutans* in dental caries progression is particularly noteworthy. The ability of *S. mutans* to enhance *Candida* adhesion and biofilm formation suggests a need to reassess caries prevention strategies, underscoring the importance of considering *Candida* species in caries risk assessment and prevention strategies.

The potential association between *Candida* infections and systemic diseases, including cancer and viral infections, is another significant finding. The ability of *Candida* species to induce inflammatory responses and modulate host immunity may contribute to tumorigenesis, particularly in oral and gastrointestinal cancers. Moreover, recent studies suggest that *Candida* biofilms may serve as reservoirs for viral pathogens, potentially exacerbating viral infections such as COVID-19. The observed decrease in salivary histatin levels in patients with COVID-19 further supports the role of saliva in maintaining oral microbial balance and highlights the need for monitoring fungal infections in patients post-COVID-19.

This review provides valuable insights into the complex interactions of *Candida* species with host immune responses and microbial communities in the oral cavity. The roles of saliva as both a protective and permissive factor in *Candida* colonization are also discussed, emphasizing the need for strategies that preserve salivary function and enhance its antimicrobial properties. Understanding the virulence mechanisms of *Candida* species, particularly biofilm formation, adhesion factors, and immune evasion tactics, is crucial for developing targeted therapeutic interventions, such as artificial saliva formulations enriched with antifungal peptides.

## Figures and Tables

**Table 1:** Differences between *C. albicans* and *C. parapsilosis*.

Feature	*Candida albicans*	*Candida parapsilosis*
Morphology	Yeast, forms true hyphae and pseudohyphae	Yeast, forms pseudohyphae but lacks true hyphae
Germ Tube Test	Positive	Negative
Chlamydospore Formation	Present	Absent
Common Infections	Oral thrush, vaginal candidiasis, systemic infections	Bloodstream infections, catheter-related infections, wound infections
Virulence	High virulence, strong biofilm formation	Lower virulence, but strong biofilm formation on medical devices
Biofilm Formation	Strong on mucosal surfaces and devices	Strong on medical devices like catheters
Enzymatic Activity	Produces proteases, phospholipases, and lipases	Produces fewer proteases and phospholipases
Resistance to Antifungals	Generally susceptible to azoles, echinocandins, and polyenes, but resistance is emerging	More resistant to echinocandins than *C. albicans*
Natural Habitat	Human mucosal surfaces (oral cavity, gastrointestinal tract, and vagina)	Skin, hospital environments, and hands of healthcare workers
Epidemiology	Most common cause of candidiasis	Common in healthcare-associated infections, especially in neonates and ICU patients
Cytokines Induced	High levels of IL-1β, IL-6, IL-17, IL-22, and TNF-α (strong inflammatory response)	Lower levels of IL-1β, IL-6, IL-17, and TNF-α, but induces IL-10 (more immunotolerant response)

Abbreviations: ICU, intensive care unit; IL, interleukin; TNF, tumor necrosis factor.

**Table 2:** Binding interactions between *Candida* species and human salivary molecules.

*Candida* Species [Reference]	Salivary Molecule	Binding Mechanism	Effect on *Candida* Species
*C. albicans* [69,71]	Proline-Rich Proteins (PRPs)	Adhesion via Bgl2p, Als1, and Hwp1	Adherence, Biofilm formation
*C. albicans* [22,75,76,77]	Mucin (e.g., MUC5B, MUC7)	Interaction with Sap2	Inhibition of hyphal formation and biofilm formation, Candidacidal activity
*C. tropicalis* *C. parapsilosis* *C. dubliniensis* [22]			
*C. albicans* [78,79,80,81,82,83,84,85]	Histatin	Binding to Ssa1/2, interaction with cell membrane	Growth inhibition, Cell membrane damage, Antifungal activity
*C. tropicalis* *C. parapsilosis* [79,81]			
*C. dubliniensis* [82]			
*C. auris* [8]			
*C. albicans* [85,89,90,91]	Statherin	Electrostatic forces	Adhesion to hydroxyapatite and epithelial cells, Transition from hyphae to yeast
*C. albicans* [92,93,94,95,96]	β-Defensin	Binding to PIP2	Fungicidal effects, Membrane permeabilization, Cell death
*C. tropicalis* [94]			
*C. parapsilosis* [94,95]			
*C. albicans* [109,110]	Lactoferrin	Binding to lactoferrin receptors	Iron sequestration, Growth inhibition, Alteration of cell wall permeability
*C. tropicalis* *C. parapsilosis* [109]			
*C. dubliniensis* [110]			
*C. albicans* [80,112]	LL-37	Binding to cell wall carbohydrates, mannan, glucan, and chitin	Transmembrane pore formation and intracellular damage
*C. tropicalis* *C. parapsilosis* [112]			
*Candida* species [106,107]	Secretory IgA (sIgA)	Binding to epitopes	Agglutination and clearance, immune evasion, inhibition of hyphal growth and virulence

Abbreviations: Sap, secretory aspartyl proteinase; PIP2, phosphatidylinositol 4,5-bisphosphate.

**Table 3:** Molecular interactions of *Candida* species in humans.

Molecule of *Candida* Species	Human Molecular Target	Function/Effect	Reference
Sap1, 2, 3, 9	Histatin 5	Inhibition of antifungal effect by degradation	[86]
Sap1, 2, 3	Complement proteins (C3b, C4b, C5)	Degradation, Inhibition of terminal complement complex formation	[114]
Sap6	Protease activated receptor (PAR) 2	Production of chemokines, Induction of neutrophil chemotaxis	[119,120]
Candidalysin	GP1bα (von Willebrand factor receptor)	Release of Dickkopf-1, Upregulation of Th17 immunity	[127]
	EGFR	Induction of immune responses	[128]
Candidalysin, Als3	EphA2/EGFR	Promotion of phagocytosis, Production of chemokines and ROS	[132]
Als3	E-cadherin, N-cadherin	Induction of endocytosis	[140]
Als1, 3, 5	Type IV collagen, fibronectin, laminin	Adhesins for invasion of hosts	[136,139]
Als proteins	CR3	Induction of inflammasome activation	[141]
Als3	Caspase-8, ASC	Activation of inflammasome	[142]
Als3, Ssa1	EGFR/HER2, E-cadherin	Promotion of adhesion and colonization	[144]
Ssa1	E-cadherin, N-cadherin	Promotion of epithelial barrier disruption	[145]
Hwp1	Fibronectin	Adhesins for invasion of hosts	[136,139]
Pra1	Complement proteins (C3, C3b, factor-H, C4BP)	Complement evasion, Enhancement of bloodstream dissemination	[1,160]
	CR3	Promotion of phagocytosis, Augmentation of tissue attachment	[159]
Mannan	Mannose receptor	Initiation of innate immune response	[167,168]
	TLR4	Initiation of inflammatory response	[15,170]
	Dectin-2	Initiation of inflammatory response, Th17 differentiation	[7,173,174]
β-glucan	Dectin-1	Initiation of inflammatory response	[15,179,180]
	TLR2	Initiation of inflammatory response	[15,182]
	EphA2	Augmentation of ROS production	[183,184]

Abbreviations: Sap, secretory aspartyl proteinase; Als, agglutinin-like sequence; Hwp1, hyphal wall protein 1; Pra1, pH-related antigen 1; EGFR, epidermal growth factor receptor; HER2, human epidermal growth factor receptor 2; EphA2, ephrin type-A receptor 2; CR3, complement receptor 3; ASC, apoptosis-associated speck-like protein containing a caspase recruitment domain; TLR, Toll-like receptor; ROS, reactive oxygen species.

**Table 4:** *Candida albicans* and oral microorganism interactions.

Oral Microorganism	Related Molecules (Bacteria/*Candida*)	Interaction Type	Reference
*Streptococcus mutans*	GtfB, GtfC/Farnesol	*S. mutans* growth, microcolony development, glucosyltransferase activity	[187]
	ComC/-	Inhibition of germ tube formation	[192]
	Mutanobactin A/-	Inhibition of yeast–mycelium transition	[193]
	SDSF/Hwp1	Inhibition of hyphal formation	[194]
	-/Als1, Hwp1	Formation of dual-species biofilm	[198]
	GtfB/Mannan	Formation of mixed-species biofilm	[199]
	GtfB/Bcr1	Promotion of *C. albicans* growth for biofilm formation	[190]
	Antigen I/II/-	Coaggregation, biofilm formation	[200]
*S. mutans, S. sanguinis*, *Actinomyces viscosus*, *A. odontolyticus*	-/Hwp1, Sap4, Sap6	Enhancement of tissue invasion and damage	[6]
*Lactobacillaceae* family	Bacteriocins/-	Competition, antifungal effects	[204]
*Lactobacillus acidophilus*	β-glucanase/β-glucan	Antifungal effects	[177]
*Porphyromonas gingivalis*	InlJ/Als3	Binding, biofilm formation	[210]
	RgpA/Als3, Mp65, enolase-1	Binding, protection against anaerobes	[208]
	-/Als1, Als3	Adherence, protection against anaerobes, gingipain activity	[209]
*Fusobacterium nucleatum*	RadD/Flo9	Binding, coaggregation	[212]
		Inhibition of hyphal morphogenesis and growth	[213]

Abbreviations: Gtf, glucosyltransferase; Hwp1, hyphal wall protein 1; SDSF, trans-2-decenoic acid; Als, agglutinin-like sequence; Sap, secretory aspartyl proteinase.

## Data Availability

No new data were created or analyzed in this study.

## Abbreviations

The following abbreviations are used in this manuscript:

Sap	Secretory asparaginyl proteinase
Als	Agglutinin-like sequence
COVID-19	Coronavirus disease 2019
Th17	Helper T cell type 17
IL	Interleukin
ROS	Reactive oxygen species
MAPK	Mitogen-activated protein kinase
PCR	Polymerase chain reaction
RFLP	Restriction fragment length polymorphism
bPRP	Basic proline-rich protein
HBD	Human β-defensin
PIP2	Phosphatidylinositol 4,5-bisphosphate
sIgA	Secretory immunoglobulin A
EGFR	Epidermal growth factor receptor
Ece1	Extent of cell elongation 1
NLRP3	Nucleotide-binding oligomerization domain-like receptor family pyrin domain-containing 3
HER2	Human epidermal growth factor receptor 2
EphA2	Ephrin type-A receptor 2
Hwp1	Hyphal wall protein 1
CFEM	Common in fungal extracellular membranes
CR3-RP	Complement receptor 3-related protein
Pra1	pH-regulated antigen 1
TLR	Toll-like receptor
TNF	Tumor necrosis factor
Gtf	Glucosyltransferase
PGE2	Prostaglandin E2
HIV	Human immunodeficiency virus
SARS-CoV	Severe acute respiratory syndrome-coronavirus
ICU	Intensive care unit
ECM	Extracellular matrix
Syk	Spleen tyrosine kinase
AP-1	Activator protein-1
AIDS	Acquired immunodeficiency syndrome
OSCC	Oral squamous cell carcinoma
OPMD	Oral potentially malignant disorder
